# Quantified Hypoxia and Anoxia in Lakes and Reservoirs

**DOI:** 10.1100/tsw.2004.5

**Published:** 2004-02-26

**Authors:** Gertrud K. Nürnberg

**Affiliations:** Freshwater Research, Baysville, Ontario, Canada

**Keywords:** oxygen depletion, anoxia, hypoxia, eutrophication, freshwater systems, climatic effects, method

## Abstract

Hypoxia and anoxia occur frequently in freshwater systems and have biological and chemical implications. *Anoxia* can be expressed and quantified as the anoxic factor; *hypoxia*, for a specific level of oxygen depletion, can be expressed as the hypoxic factor in lakes, reservoirs, and river sections. These methods summarize information of individual dissolved oxygen profiles as annual values or factors that facilitate comparison between and within lakes. Therefore, these factors are useful in the formulation and testing of hypotheses related to the dissolved oxygen status in water bodies. Methods of calculating different factors for different oxygen levels and water layers, including those applying separately to the epilimnion and hypolimnion, are presented in detail. Proven and potential applicability include: (1) the quantification of relationships with lake water quality variables and lake classification (trophic state), (2) the evaluation of restoration techniques with respect to their effects on hypolimnetic oxygen depletion, (3) the determination of internal phosphorus loading in stratified and polymictic lakes, (4) the exploration of habitat constraints due to hypoxia (e.g., fish species richness and winterkill), (5) forecasting potential effects of climatic change on oxygen content and internal phosphorus loading, and (6) the establishment and examination of criteria and guidelines with respect to hypoxia by custom-made definitions.

## INTRODUCTION

Hypoxia and anoxia occur frequently in freshwater systems[1], and increases in organic and nutrient loading and changes in water flow have increased oxygen depletion in lakes[2] and reservoirs[3]. Oxygen depletion is so widespread that methods of remediation have been proposed and applied in Europe and North America for many years[4,5,6].

Ways to quantify oxygen depletion in lakes were introduced before including oxygen depletion rates[7], the probability of anoxia[8], anoxic factor (AF)[1], and hypoxic factor (HF)[9]. Oxygen depletion rates are determined from decreasing dissolved oxygen (DO) concentrations before the onset of anoxia. They do not describe the extent of anoxia in lakes, but only the speed of acquiring anoxia and therefore do not quantify anoxia itself. The probability of anoxia was developed from regression analysis of 55 stratified lakes with oxic, slightly anoxic, and considerably anoxic hypolimnia using lake characteristics pertaining to morphometry, hydrology, and nutrient load. This measure is based on an empirical model and therefore is restricted by the characteristics of the lakes for which the model was formulated; it appears to underestimate anoxia in pristine, oligotrophic lakes that may be anoxic for reasons other than high productivity, as noted in [1].

In comparison, the anoxic or hypoxic factors quantify extent and duration of anoxia or hypoxia because they are computed from numerous measured DO profiles taken throughout the year[1,9]. The annual values of the factors are comparable between and within lakes and hence facilitate the formulation and testing of hypotheses related to lake oxygen. Furthermore, the factors are useful in setting DO criteria and guidelines because the quantification of hypoxia is based flexibly on individual threshold values and can be adapted to various DO levels and spatial restrictions (e.g., mixed surface layer only). Presented here is a comprehensive summary of the methods used to calculate both factors, examples of their proven and potential applicability, and their flexibility in dealing with differing DO thresholds and criteria.

## DEFINITIONS AND COMPUTATION

The anoxic and hypoxic factors quantify the extent and duration of anoxia and hypoxia. They are based on a series of measured oxygen profiles and morphometric (hypsographic) data and can be computed for any water body (lake, reservoir, river, estuary, or marine area).

The difference between HF and AF lies in the application of a different minimum or threshold DO concentration in the calculation procedure. The AF is based on the oxycline depth or its approximation (i.e., the depth at which 1 or 2 mg $L^{-1}$ DO is observed), while the HF is based on a defined threshold that indicates the hypoxic criterion of choice for that particular water body, e.g., 6.5 mg $L^{-1}$ DO. Both factors are computed for the hypothetical lake in Fig. 1 according to the following steps:

1. For AF, the oxycline is determined at 1 or 2 mg $L^{-1}$ DO concentration from (DO) profiles. The choice of the threshold values depends on the method of DO measurement. For example, 2 mg $L^{-1}$ measured by a DO probe about 1 m above the sediment usually coincides with anoxic conditions at the sediment surfaces located at that depth. For HF, the depth of the DO concentration threshold in question, e.g., 6.5 mg $L^{-1}$, is determined.
2. The periods (total number *n*) are established for which the DO levels are at approximately the same depth according to the chosen threshold.
3. The duration of each period of constant DO levels ($t_i$, in days) is multiplied by the corresponding area ($a_i$) and divided by the total surface area ($A_{oi}$) for the period (*i*). Especially in reservoirs where volumes and therefore areas change, it is important to use the surface area specific for the period. In water bodies without large volume changes, the average surface area $A_o$ can be used instead. Correction for surface area is done to render this index comparable across waters similar to other areal measures, e.g., areal nutrient loads and fish yield.
4. These *n* terms, numbers of periods at different oxyclines, are then added up over the season or year, according to Eq. 1.

$$AF\ or\ HF = \frac{t_1 \cdot a_1}{A_{o1}} + \frac{t_2 \cdot a_2}{A_{o2}} + \frac{t_3 \cdot a_3}{A_{o3}} + ... + \frac{t_n \cdot a_n}{A_{on}} = \sum_{i=1}^{n} \frac{t_i \cdot a_i}{A_{oi}} \quad (1)$$

Expressed this way, AF or HF is the sum of ratios and represents the number of days in a year or season that a sediment area equal to the (lake) surface area is anoxic or hypoxic.

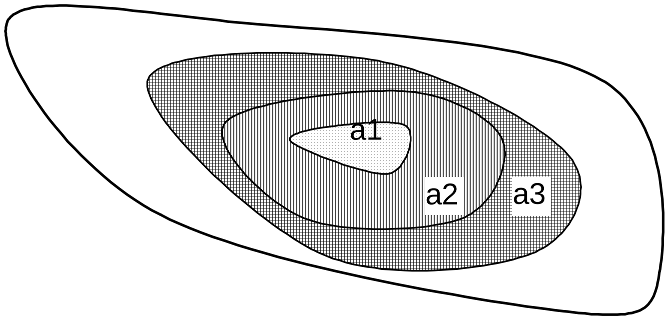

**FIGURE 1**. The sketch indicates different areas of a hypothetical lake that become anoxic sequentially with time, where *a1* becomes anoxic first, then *a2*, etc.

## Seasonal Factors

When AF and HF are determined for the stratification periods separately, a winter (e.g., $AF_{win}$ in d winter$^{-1}$) or summer factor (e.g., $AF_{sum}$ in d summer$^{-1}$) is specified. When there is no winter anoxia, $AF_{win}$ is zero and $AF_{sum}$ is equal to AF in d yr$^{-1}$.

## Epilimnetic Factors

To consider the mixed surface layer separately, epilimnetic factors ($AF_{epi}$, $HF_{epi}$) can be computed. These factors are determined by subtracting, for each period *i*, terms corresponding to anoxia or hypoxia in the seasonal stratified area below the thermocline ($a_{thermo\_i}$) from those for the whole water column before summation, according to Eq. 2.

$$AF_{epi} \text{ or } HF_{epi} = \sum_{i=1}^{n} \left( \frac{t_i \cdot a_i}{A_{o\_i}} - \frac{t_i \cdot a_{thermo\_i}}{A_{o\_i}} \right) \qquad (2)$$

Such spatially distinct factors are especially useful in determining certain habitat requirements and for the support of guidelines specific to the mixed zone. For example, occasional epilimnetic anoxia may lead to a complete fish kill, while hypolimnetic anoxia may (only) prohibit the occurrence of certain fish species (especially coldwater species like Salmonidae, Gadidae, and Corigonidae).

## End of Summer Stratification

In stratified lakes, much of the oxygen depletion occurs in the fall when DO profiles may not be available. In this case, the endpoint at fall turnover can be predicted by a model based on summer average hypolimnetic temperature, mean depth, and latitude from 92 worldwide lakes according to Eq. 3, $R^2$ = 0.47, $p < 0.0001$[10].

$$\text{Log (fall turnover date)} = 2.62 - 0.116 \log (T) + 0.042 \log (z) - 0.002 \text{ Latitude} \qquad (3)$$

where: fall turnover date, Julian day of the year; T, average July–August temperature at ca. 1 m above the bottom at the deepest location of the lake (C°); z, mean depth, i.e., lake volume/lake surface area (m).

## Method Precision

The precision of the AF calculations was estimated by comparing AF computations by two different individuals for weekly to monthly DO profiles over 88 (accumulated) years of seven lakes, and their long-term averages of up to 11 years per lake. The error was 8% of the mean for the individual years, but differences between computations were not significant for the long-term averages.

## Method Accuracy

The estimates are more accurate if they are based on DO profiles from more than the deepest location. Nürnberg[11] observed that in two lakes with long complicated shapes, anoxia started and ended earlier at the shallower sites. *Spatial within-lake variability* is discussed in more detail in the section on Application.

## Example Brownlee Reservoir

Fig. 2 presents DO isopleths for the deep section of the Snake River Brownlee Reservoir in 1999 and 2000. The factors computed from these data were as follows: AF was 84 and 106 d $yr^{-1}$ in 1999 and 2000, while HF was 135 and 175 d $yr^{-1}$[12]. Epilimnetic factors were 7 and 8 d $yr^{-1}$ $AF_{epi}$ and 43 and 60 d $yr^{-1}$ $HF_{epi}$ for 1999 and 2000.

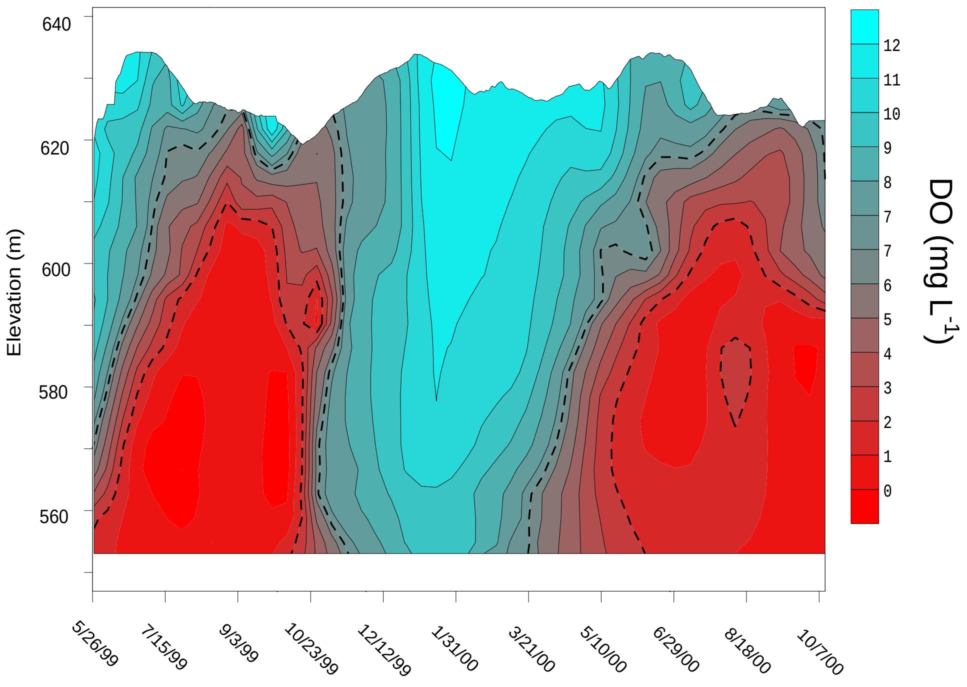

**FIGURE 2**. DO isopleths in Brownlee Reservoir for 1999 and 2000 near the dam in the lacustrine section. Broken lines represent thresholds at 2.0 mg $L^{-1}$ as used for the anoxic factor and 6.5 mg $L^{-1}$ for the hypoxic factor. (From [9], reprinted with permission.)

### Determination of AF from One End-of-Summer Sampling Event

The redox potential can be used to indicate the severity of anoxia once the water is anoxic, although its exact value is largely influenced by the amount of iron. Consequently, the redox potential measured within 1 m above the sediment surface at the end of the anoxic summer period and a variable related to iron (iron concentration of the sediment or lake water) was highly significantly correlated with AF in several Ontario lakes; it predicted AF of the previous summer reasonably well ($n = 19$, $R^2 = 0.76$ or $n = 52$, $R^2 = 0.50$[1]). Therefore, such relationships may be useful in estimating AF values from one sampling effort in remote and poorly studied lakes.

## APPLICATION

Applications for methods of quantifying oxygen depletion are obvious. Because anoxic and hypoxic conditions can be expressed as a single annual value and be compared between and within lakes for different years, the formulation and testing of hypotheses related to lake oxygen is immensely facilitated. Since AF and HF are based on a large quantity of observed and measured data, they are more robust and exact than other estimates like rates of oxygen depletion, modeled measures (e.g., probability of anoxia and end-of-summer DO profiles), and simple DO criteria (i.e., threshold concentrations) as discussed in Nürnberg[1]. Nonetheless, the AF can be predicted with reasonable accuracy from models containing nutrient and morphometric variables. The following applications have been found most useful.

### Spatial Within-Lake Variability

In large and morphometrically distinct water bodies, the factors can be estimated separately for different sections. For example, AF was quite different between the two basins in small (77 ha) Cinder Lake, Ontario, at 12 and 40 d summer$^{-1}$. Similarly pronounced were differences for the 100-km long, narrow (maximum 1-km wide) reservoir, Brownlee Reservoir on the Snake River, Idaho–Oregon[12]. The total reservoir had an AF of 68 d yr$^{-1}$ on average between 1962 and 2001, ranging from 53–90 d yr$^{-1}$. But AF of the 48-km long, deep lacustrine section was higher, 88 d yr$^{-1}$ on average, ranging from 72–118 d yr$^{-1}$, because this section is stratified all summer long (Fig. 3). On the other hand, AF for the 52-km long, shallow riverine section was only 10 d yr$^{-1}$ in 1999 and 14 d yr$^{-1}$ in 2000, because it is often mixed and reaerated with atmospheric oxygen. Epilimnetic factors were smaller than factors for the whole water column, as expected (Fig. 3). Severe anoxia in this reservoir is apparent since the long-term average lacustrine $AF_{epi}$ was 10 d yr$^{-1}$, indicating that overall, an area in the lacustrine epilimnion equal to the lacustrine surface area is overlain by water below 2 mg L$^{-1}$ DO for 10 d summer$^{-1}$. The average $HF_{epi}$ of 58 d yr$^{-1}$ indicates that the Oregon State criterion of 6.5 mg L$^{-1}$ DO for the water column was exceeded in the lacustrine epilimnion for an equivalent of ca. 2 months on average.

### Annual Variability

Year-to-year variability can be quite large and ranged from 72–118 d yr$^{-1}$ AF and 102–215 d yr$^{-1}$ HF in lacustrine Brownlee Reservoir between 1962 and 2001 (Fig. 3); from 45–68 d yr$^{-1}$ AF in a small urban kettle lake, Lake Wilcox, between 1987 and 2002; and from 0–10, 9–30, and 10–50 d yr$^{-1}$ AF in three small glacial lakes on the Precambrian shield[Nürnberg, unpublished data,1,5,9].

Physical characteristics, especially hydrology in flow-managed reservoirs, have been found to control a significant portion of the between-years variance of DO depletion. For example, several annual and seasonal hydrological variables were significantly ($p < 0.01$) correlated with AF, HF, $AF_{epi}$, and $HF_{epi}$ in Brownlee Reservoir. In particular, spring (April–May), summer inflow (July–Sept), and late fall (Oct–

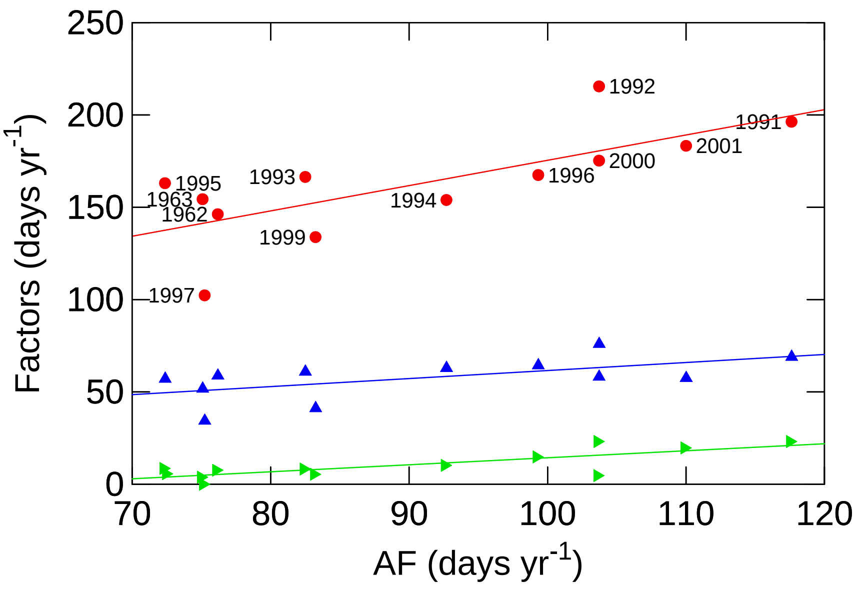

**FIGURE 3**. Comparison of AF (x-axis) with several other factors for the lacustrine section of Brownlee Reservoir, Idaho–Oregon: HF (circle, years are indicated), $HF_{epi}$, (triangle), and $AF_{epi}$ (arrow). All regressions are significant at least at $p < 0.04$ for n = 12 (lines are shown). Data from [9] and Nürnberg unpublished.

Dec, Fig. 4 A, B) flushing rate or its inverse, the water residence time, were strongly correlated with the factors, so that they were smallest during periods of high inflow or flushing[9]. Such a good relationship of the fall outflow or residence time with anoxia and hypoxia can be expected, because severe DO depletion happens mostly in the summer and fall; low flow rates out of the dam and high residence times would stabilize stratification in the lacustrine section, leading to increased hypoxia. On the other hand, a strong inverse correlation for spring inflow with hypoxia can be explained by a delay in the onset of stratification.

## Evaluation of Lake Restoration Techniques Aiming at the Reduction of Oxygen Depletion

Hypolimnetic oxygenation and aeration were used for 3 years, in an effort to improve water quality of Lake Wilcox[13]. Although fall turnover was earlier during treatment years than ever recorded before, AF was as high as and higher than in seven pretreatment and two post-treatment years, rendering the benefit of the aeration treatment questionable.

Hypolimnetic withdrawal, the preferential discharge of hypolimnetic water by damming the surface water, has been used to enhance the export of nutrients and reduced substances out of lakes. The oxygen conditions, expressed as AF, indicate that the decrease in hypolimnetic anoxia was slower than the decrease of epilimnetic nutrient concentration and algal biomass. A positive effect on AF was evident only after at least four treatment years, while epilimnetic phosphorus concentration decreased already in the first year after operation in Lake Wononscopomuc, Connecticut[14].

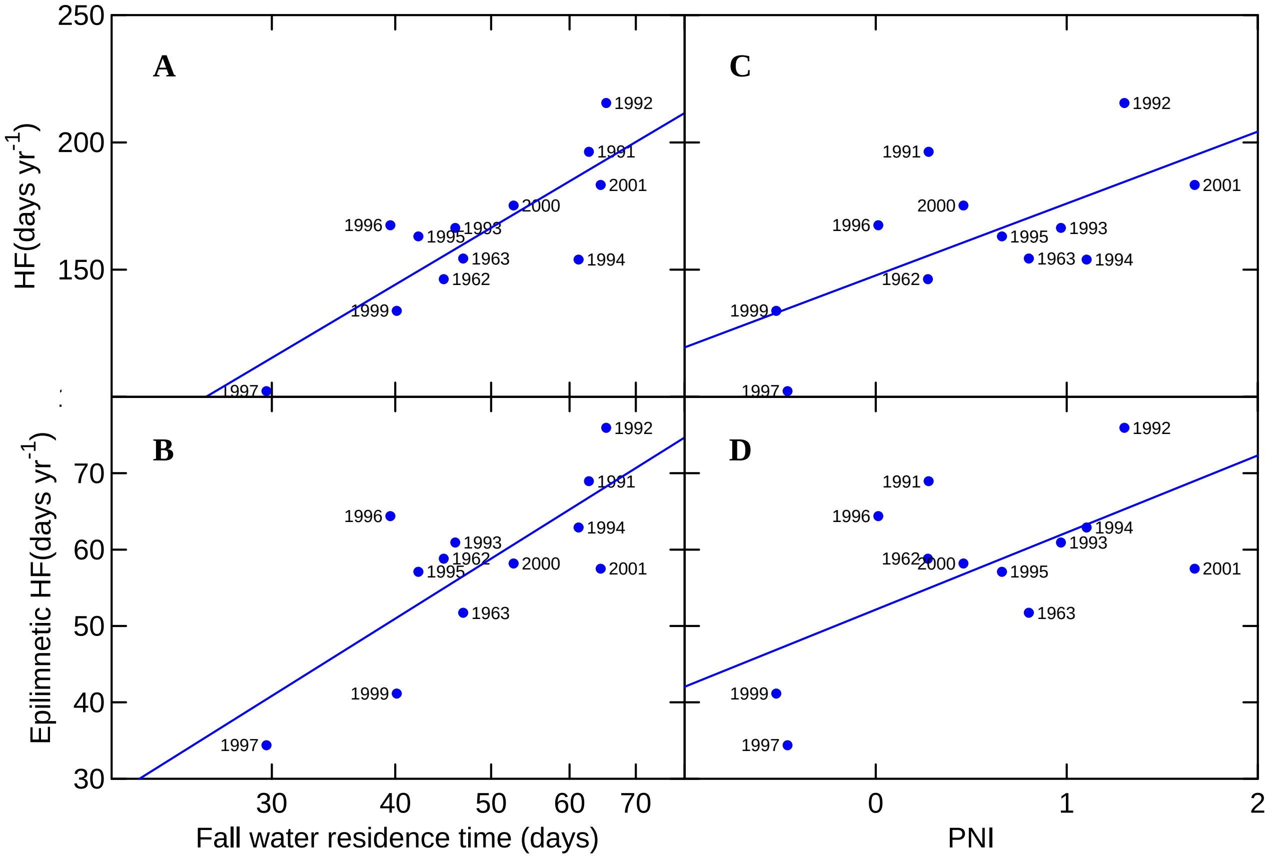

**FIGURE 4.** Lacustrine HF (top) and $HF_{epi}$ (bottom) of Brownlee Reservoir vs. fall (October to December) water residence time (total of 92 days, left panel) and the Pacific Northwest Climate Index (PNI, right panel). Regressions are significant; at least at $p < 0.005$ for the log-transformed water residence time and at $p < 0.04$ for the PNI relationships (n = 12, regression lines are shown). Revised from [9] including unpublished data.

## Lake-to-Lake Variation

Anoxia can vary extensively between lakes and quantification of anoxia by the AF has been proven useful in explaining lake-to-lake variation. Even in a geographically close area like the Muskoka–Haliburton region on the Precambrian shield in southern Central Ontario, AF ranged from 0–70 d $yr^{-1}$ and at least 50% had AF > 10 d $yr^{-1}$. These lakes are relatively pristine, often colored, and usually small, but deep. Occasional anoxia in many of these remote lakes is probably due to a combination of morphometry and high organic acid content due to natural humic and fulvic acids exported from surrounding wetlands. Mesotrophic and eutrophic lakes in more fertile regions, like in the St. Lawrence–Great Lakes region had consistently high AF between 40 and 70 d $yr^{-1}$. Such differences in lakes and their AF reflect differing water and catchment chemistries and indicate the need for diverse management strategies[5].

Of all tested physical and chemical variables (including dissolved organic carbon as measure of organic acids that contributed only 17% to the variance of AF[1]), a dependency of anoxia on nutrient concentration and morphometry was found the most important. Accordingly, multiple regressions of long-term average AF on average lake TP and a morphometric ratio, measured as $z/A^{0.5}$ (m $km^{-1}$), were highly significant in North American lakes[1] (Table 1, Eq. A, B). Recent analysis with data from Nürnberg[15], showed a similarly strong relationship with long-term average summer total nitrogen (Fig. 5, Table 1, Eq. C). These long-term relationships can be used to model (or predict) AF in lakes where DO

**TABLE 1**
**Relationships and Models Involving AF in North American Stratified Lakes, Where Data of Equations E to G are Based on Central Ontario Lakes**

| Eq. | Predicted[1] | Regression Eq.[1] | N | $R^2$ | Source |
|---|---|---|---|---|---|
| A | $AF_{sum}$ | $-35.4\ (5.1) + 44.2\ (4.3) \log (TP_{annual}) + 0.950\ (0.187)\ z/A_o^{0.5}$ | 73 | 0.65 | [1] |
| B | $AF_{sum}$ | $-36.2\ (5.2) + 50.1\ (4.4) \log (TP_{summer}) + 0.762\ (0.196)\ z/A_o^{0.5}$ | 70 | 0.67 | [15] |
| C | $AF_{sum}$ | $-173\ (22) + 73.0\ (8.6) \log (TN_{summer}) + 0.925\ (0.272)\ z/A_o^{0.5}$ | 54 | 0.62 | This study |
| D | $AF_{sum}$ | $-39.9\ (9.7) + 27.0\ (4.0) \log (\text{TP-Load})$ | 17 | 0.76 | [1] |
| E | Fish species number[1] | $0.97\ (2.42) - 1.53\ (0.49) \log (AF_{sum}+1) + 5.38\ (1.02) \log (A_o)$ | 52 | 0.51 | [20] |
| F | Fish species number[1] | $4.92\ (1.38) - 6.12\ (1.44) \log (AF_{win}+1)^* + 0.56\ (0.11)\ z$ | 32 | 0.71 | [20] |
| G | Winterkill-$AF_{win}$ | $-1 + 10^{(0.091\ z + 0.804)}$ | — | — | Based on Eq. F |

*Note*: All regressions and partial probabilities are highly significant at $p < 0.001$, except in Eq. F where noted with *, $p < 0.01$. Standard errors of the regression coefficients are given in parenthesis.

1 Variables: $AF_{sum}$, summer AF (d summer$^{-1}$); $AF_{win}$, winter AF (d winter$^{-1}$); Winterkill-$AF_{win}$, value of $AF_{win}$ above which a fish winterkill is likely for given z; z, mean depth (m); $A_o$, lake surface area (km$^2$); $TP_{annual}$, annual water-column average TP (µg L$^{-1}$); $TP_{summer}$, epilimnetic summer TP (µg L$^{-1}$); $TN_{summer}$, epilimnetic summer TN (µg L$^{-1}$); Fish species number, also called fish species richness, number of species of all fish present in specific lake.

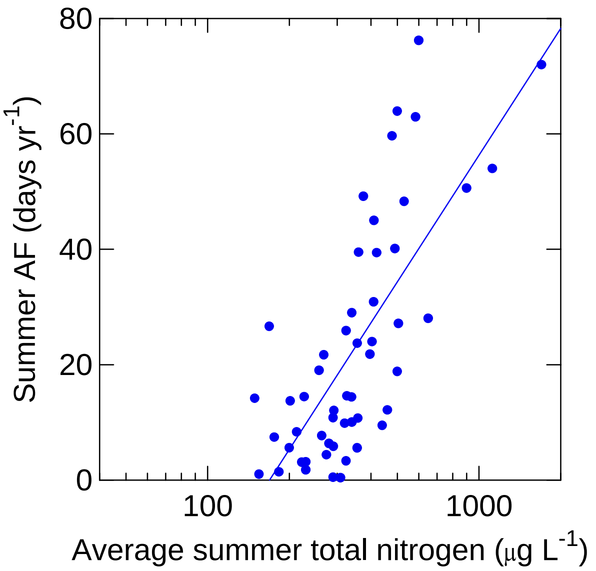

**FIGURE 5**. Summer AF in 54 dimictic lakes of northeastern North America vs. long-term averages of summer total nitrogen concentration. Regression line of Eq. C in Table 1 is shown, where one outlier was removed and only AF above zero was included.

data are not available or difficult to obtain, like in polymictic lakes. Since AF describes the sediment surface that is overlain by anoxic water in stratified lakes, it can be hypothesized that its predicted value resembles anoxic sediment surfaces in polymictic lakes. Such a modeled variable is useful in determining internal load for shallow lakes as described below, where release rates may be available but not the extent of anoxic sediment areas.

## Trophic State Classification

Because of the dependency on nutrient concentration, the anoxic factor was used to establish limits for trophic state classification with respect to anoxia[15]. Because colored lakes often have anoxic hypolimnia despite low algal biomass, the classification of data including 70 North American lakes indicates that oligotrophic lakes can have an AF of up to 20 d $yr^{-1}$. Consequently, when classified with respect to trophic state, an AF below 20 d $yr^{-1}$ indicates oligotrophic conditions, 20–40 d $yr^{-1}$ are usually found in mesotrophic lakes, 40–60 d $yr^{-1}$ represent eutrophic conditions, and above 60 d $yr^{-1}$ is typical for hypereutrophic conditions. A more exact measure of trophic state would include the morphometric ratio, because the deeper the lake compared to its area (the larger $z/A^{0.5}$), the larger the AF. From Eq. B, Table 1, a chart was drawn to assist the evaluation of a specific trophic state with respect to anoxia (Fig. 6).

## Internal Load Calculation

The anoxic factor was originally developed to determine internal phosphorus load in stratified lakes as the product of anoxic areal release rates (experimentally determined or predicted from sediment phosphorus concentration) and AF according to Eq. 4[11].

$$\text{Internal load} = \text{Release rate} \cdot AF_{sum} \qquad (4)$$

where: (summer) internal load, mg total phosphorus (TP) per lake surface area ($m^2$) per summer, and phosphorus release rate representing a summer average, in mg TP released per anoxic sediment area per day of the anoxic summer period (mg $m^{-2}$ $d^{-1}$). If there is phosphorus release under ice in the winter, a release rate representative for the winter and $AF_{win}$ are substituted in Eq. 4 to estimate winter internal load.

Modeling and prediction of future internal load and subsequently lake phosphorus concentration and trophic state can be accomplished by predicting one or both of its components, AF and phosphorus release rates, according to Eq. 4. For example, a sequence of models and empirical equations predicted future water quality after the potential increase in lakeshore development of a seasonal community in the reach of the metropolitan Toronto (ca. 500 lakes in the District of Muskoka[16]). In particular, future TP concentrations based on increased external loading (i.e., a minimum estimate of TP concentration since internal load is not being considered) were used to predict future anoxic factors (from equations in Table 1 and release rates based on regional release rate - TP relationships). Postdevelopment internal load could then be computed as the product of these variables (Eq. 4) and used to predict annual average lake TP concentration and other water quality variables like chlorophyll and Secchi transparency[17].

As mentioned previously, of particular usefulness is the application of the AF model to shallow lakes with intermittent stratification. The direct determination of internal load in these polymictic lakes is almost impossible without major technical effort (determination of detailed hydrologic and phosphorus mass balance, including down and upward fluxes throughout the summer), since external mix with internal phosphorus inputs frequently and untraceably throughout the summer[18]. However, a modeled AF can be used to determine internal load according to Eq. 4 in shallow, polymictic lakes, where release rates may be measured in the laboratory or predicted from sediment phosphorus content[19], but the extent and duration of sediment anoxia is unknown.

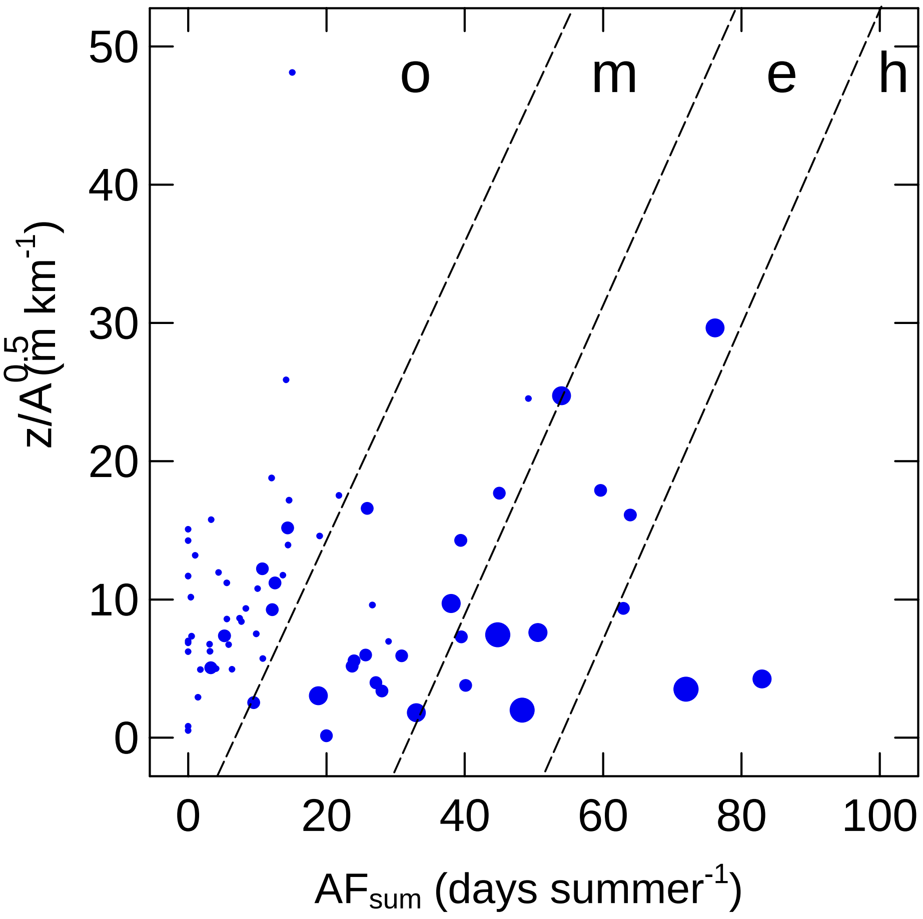

**FIGURE 6.** Summer AF ($AF_{sum}$) and the morphometric variable $z/Ao^{0.5}$ can be used to classify lakes with respect to their trophic state. Observed values from 70 North American lakes are presented[15]. Symbol sizes indicate the four trophic states oligotrophy, mesotrophy, eutrophy, and hypereutrophy (from small to large) and lines indicate the trophic state boundaries based on $TP_{summer}$ in Eq. B, Table 1.

## Fish Species Richness

Fish species richness was found to be correlated with AF in Central Ontario lakes after variability due to lake area was taken into account, and winter kill could be predicted by using an $AF_{win}$ that quantifies anoxia under ice, and mean depth (Table 1, E–G). In particular, cold water species including Salmonidaes, Coregonidae, and Gadidae were sensitive to summer and winter anoxia and they only occurred when $AF_{sum}$ was below 32 d summer$^{-1}$ or $AF_{win}$ was below 4.4 d winter$^{-1}$[20].

## Climatic Effects

Since the factors quantify annual trends, climatic effects on DO depletion can be investigated. For example, the Pacific Northwest Index (PNI) was significantly correlated with HF and $HF_{epi}$ in Brownlee Reservoir (Fig. 4 C, D)[9]. The PNI is a normalized index based on three terrestrial climate variables for the western North American continent: the air temperature at San Juan Islands, the total precipitation at Cedar Lake in the Cascade Mountains, and snowpack depth on Mount Rainier, Washington State[21].

### Establishment of Criteria and Guidelines

Hypoxia causes species changes in fish[20,22] and in macro benthos[23,24], so that water quality objectives have been established by governmental agencies to restrict the acceptable hypoxic level. Typically, a threshold DO concentration is set under which a water body is considered to be "impaired" and a plan is initiated to attain the guidelines, including the process of determining a total maximum daily load (TMDL) in the U.S.[25]. In the case of Brownlee Reservoir as presented here, this threshold is 6.5 mg $L^{-1}$. However, these levels vary for different water bodies, agencies, and intended usage between 4.8 and 10.5 mg $L^{-1}$[9], so that a useful quantification of hypoxia must be flexible and adaptable respective these thresholds, as can be accomplished by specific definitions of the hypoxic factor.

### Additional Applications

AF helped to compare methods to measure denitrification rates in lakes and determine the most appropriate one[26] and reveal the dependency of hypolimnetic anoxia on depth and volume in tropical African reservoirs[27]. The anoxic factor and its relationship with phosphorus and morphometry were combined with information on fossil midge (Chironomidae) assemblages in paleolimnological studies to establish training sets and hindcast hypolimnetic anoxia for the past[23,28,29]. In this way specific hypotheses could be supported, for example, that AF is correlated to the average end-of-summer hypolimnetic oxygen concentration and that the damming of lakes during European settling affected the oxygen status of lakes[30].

## CONCLUSIONS

It can be concluded that the quantification of anoxia and hypoxia leads to increased knowledge of the controlling mechanisms of oxygen depletion in water bodies. Although it has not been applied to rivers other than run-of-the-river reservoir sections, it should provide a useful quantification of hypoxia and anoxia there as well, since hypoxia is widespread in these freshwater systems[3,31,32]. The concept could be especially useful in estuarine and marine systems, since hypoxic conditions are widely spread in saline environments. Especially in recent years, anthropogenic impacts have led to severe increases in estuarine and coastal anoxia (e.g., Gulf of Mexico[33], European coast lines[34]). For example, Diaz[35] describes 44 marine areas of moderate to severe hypoxia world-wide.

## ACKNOWLEDGMENTS

Helpful comments by the editor of Freshwater Systems, Karl Havens, and several referees including Jack Jones, are acknowledged. Bruce LaZerte provided constructive and supportive critique. The concept of the *hypoxic* factor was developed while working with Brown and Caldwell for the City of Boise, ID on the Snake River Hells Canyon TMDL (total maximum daily load).
